# Preschool children in Danish out-of-hours primary care: a one-year descriptive study of face-to-face consultations

**DOI:** 10.1186/s12875-019-0922-y

**Published:** 2019-02-26

**Authors:** Jørgen Lous, Grete Moth, Linda Huibers, Peter Vedsted, Morten Bondo Christensen

**Affiliations:** 1 Research Unit for General Practice, Department of Public Health, University of Southern Denmark, Jagtvej 20A, DK-8270 Højbjerg, Denmark; 2 Research Unit for General Practice, Aarhus University, Aarhus, Denmark; 3 Research Unit for General Practice & section for General Practice, Department of Public Health, Aarhus University, Aarhus, Denmark

**Keywords:** After-hours care, Reason for encounter, Diagnosis, Drug prescriptions, Anti-bacterial agents, Patient satisfaction

## Abstract

**Background:**

The demand for out-of-hours (OOH) primary care has increased during the last decades, with a considerable amount of contacts for young children. This study aims to describe the reasons for encounter (RFE), the most common diagnoses, the provided care, and the parental satisfaction with the general practitioner (GP) led OOH service in a Danish population of children (0–5 years).

**Methods:**

We conducted a one-year cross-sectional study based on data for 2363 randomly selected contacts concerning children from a survey on OOH primary care including 21,457 patients in Denmark. For each contact, the GPs completed an electronic pop-up questionnaire in the patient’s medical record. Questionnaire items focussed on RFE, health problem severity, diagnosis, provided care, and satisfaction. The parents subsequently received a postal questionnaire.

**Results:**

The most common RFE was non-specific complaints (40%), followed by respiratory tract symptoms (23%), skin symptoms (9%), and digestive organ symptoms (8%). The most common diagnosis group was respiratory tract diseases (41%), followed by general complaints (19%) and ear diseases (16%). Prescriptions were dispensed for 27% of contacts, and about ¾ were for antibiotics. A total of 12% contacts concerned acute otitis media; antibiotics were prescribed in 70%. A total of 38% of contacts concerned fever, and ¼ got antibiotics. A total of 7.4% were referred for further evaluation. The parental satisfaction was generally high, but 7.0% were dissatisfied. Dissatisfaction was correlated with low prescription rate.

**Conclusion:**

Respiratory tract diseases were the most common diagnoses. The GPs at the OOH primary care service referred children to hospital in 7.4% of the face-to-face consultations, and the provided care was evaluated as non-satisfying by only 7.0% of the parents. Clinical implications of the findings mean room for less prescription of antibiotic to children with ear diseases and a need for research in factors related to dissatisfaction.

## Background

The out-of-hours (OOH) primary care service has been used increasingly during the last decades. At the same time, the organization of the OOH service has changed in many countries: small rotation groups have become large-scale general practitioner (GP) cooperatives, and telephone triage performed by GPs or nurses has become an essential part of the healthcare system [1, 2].

In the Central Denmark Region (CDR), patients in need of acute care outside office hours must call the OOH primary care service, where GPs answer the calls and perform telephone triage. The GPs can end the call by giving advice or prescribing medication (telephone consultation), triaging to a face-to-face consultation with a GP (clinic consultation or home visit), or referring the patient directly to a hospital (emergency department or paediatric department). A substantial number of the calls to the OOH service concern children aged 0–5 years, and the children often have symptoms of infectious disease [3, 4]. It has been widely discussed if all children attending the OOH service should be seen by a paediatrician or by a GP, who serves as a gatekeeper to secondary care [5]. However, little is known about RFE and parental satisfaction in children seen in OOH primary care.

The objective of this study was to describe face-to-face consultations for children aged 0 to 5 years in OOH primary care, specifically the RFEs, the diagnoses recorded by the triaging and treating GPs, and the provided care in terms of dispensed prescriptions, reason for referral, and parental satisfaction with the contact.

## Methods

### Study design and setting

The present population-based cross-sectional study is based on data from a random sample of patient contacts to the OOH primary care service in the CDR, which is one of five Danish regions. These data were collected from June 2010 to May 2011 as part of the OOH care cohort study referred to as ‘the LV-KOS study’ [6, 7]. The organization of OOH primary care has not been changed since data collection.

Danish GPs provide regional OOH primary care on a rotating basis. The OOH primary care service in the CDR covers a population of 1.2 million citizens, and the service consists of two call centres and 13 consultation centres located throughout the region. Opening hours are 4 pm - 8 am on weekdays and all day on weekends and public holidays. The OOH registration system is fully computerised, and each contact is registered in the patient’s medical record through the unique civil registration number assigned to every Danish citizen. An electronic copy of the record is subsequently sent to the patient’s own GP, and the data are transmitted to the regional administration for remuneration purposes as GPs are paid according to a fee-for-services model [6].

### Data collection and variables

The electronic OOH registration system provided data on patient age and gender, date and time of contact, type of contact, the GP’s clinical notes, and detailed prescription information [7]. All prescriptions were automatically registered in the system using the Anatomical Therapeutic Chemical (ATC) classification System.

**Fig. 1:**
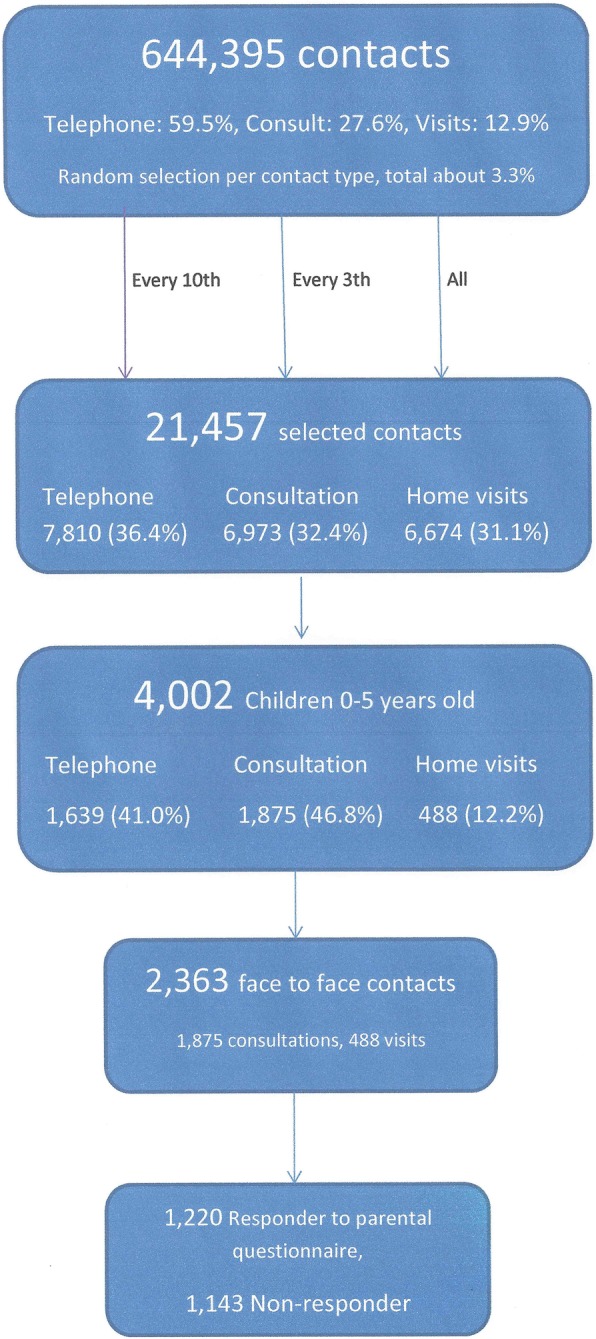
Flow diagram of study population selection. Note: For one GP on duty per type of shift, the computer system randomly selected contacts (every 10th telephone consultation, every 3rd clinical consultation, and all home visits)

Additionally, a pop-up questionnaire completed by participating GPs were used to collect extra data. For one GP on duty per type of shift, the computer system randomly selected contacts (every 10th telephone consultation, every 3rd clinic consultation, and all home visits) for inclusion in the LV-KOS study (Fig. 1) [7]. In this way, all included contacts were chosen by random within their contact type. Development of the GP questionnaire involved cognitive interviews of 12 GPs to improve the face validity of the questionnaire and a pilot test, which resulted in minor changes. The questionnaire addressed issues such as severity of health problem, diagnosis, and provided care [6].

Two-tree days after the contact with the OOH service, a postal questionnaire was sent to the parents of the included 2363 children; 1223 (51.8%) questionnaires were completed and returned. The questionnaire focused on their experience with the OOH service [6, 7]. Parents were asked to rate the perceived severity of the health problem, the duration of symptoms, and their satisfaction with the OOH service. Only these three variables from the parental questionnaire were included in this study.

We used the International Classification of Primary Care, second edition (ICPC-2), to code RFE based on the GP’s clinical notes and diagnosis [8]. The main (first mentioned) RFE was designated ‘primary RFE’ and others ‘secondary’. Secondary RFE was only recorded if it had another code number or chapter number than the primary RFE. The RFE was written in the patient’s medical record by the GPs. Coding was performed by a specially trained medical student who also received supervision from one of the authors.

### Population

During the one-year study period, 644,395 contacts were registered in the OOH service in the CDR (59.5% telephone contacts, 27.6% face-to-face consultations, and 12.9% home visits). The LV-KOS study included 21,457 contacts, i.e. 3.3% of all contacts to the OOH service (Fig. 1). The inclusion and exclusion criteria have been described earlier [6, 7]. Due to variations in the pop-up interval of registrations, the distribution of registered contacts in the LV-KOS is not comparable to the distribution of all contacts to the OOH service. Each patient could be included more times during the one-year study period. Thus, the risk of including an already included patient was about 1:30 when the patient contacted the OOH service a second time [7]. For this study, we included children from 0 to 5 years of age, in this paper called children. Because of no objective information on the diagnosis the telephone contacts are not included in the detailed analysis in this study.

### Statistics

We generated simple descriptive statistics by using IBM SPSS version 24 and the “Statistics with confidence” programme, 2nd edition, which provided a 95% confidence interval (CI) and a two-sided *p*-value of 5% [9]. Because several variables were on an ordinal scale, Spearman’s rho rank-order correlation coefficient was used to calculate rank-order correlation between variables due to non-normal distribution. The rho value ranges from − 1 to + 1, and zero indicates no linear association between two variables.

## Results

Of the 21,457 contacts, 36.4% were telephone contacts, 32.4% were clinic consultations, and 31.1% were home visits [6]. A total of 4002 contacts concerned children (aged 0–5 years), and 1639 (41%) of these were telephone contacts (Fig. 1). The present study focuses on the 2363 contacts concerning children who were seen by a GP at a face-to-face consultation in the OOH clinic (*n* = 1875) or at a home visit (*n* = 488).

A completed questionnaire was returned by 1220 of the parents (51.6%). A non-respondent analysis showed only minor differences between the respondents and non-respondents. The GPs more frequently rated non-respondents not to be seriously ill (70.3%) compared to respondents (65.2%) (difference: 5.1%, CI: 1.3–8.8%).

In Table 1 contact types along with gender and age of the included children and parental assessment are listed together with the GPs’ assessment of health problem severity, and the parents’ estimated duration of symptoms, severity, and satisfaction with the contact. Moreover, prescription rates (ranged from 0 to 37.7%) and the rates association for each variable are presented.

**Table 1:** Contact type, characteristics of participants, GP- and parent-assessed of severity, duration of symptoms, parental satisfaction, and prescription rates in preschool children seen by a GP in a face-to-face consultation at the out-of-hours service

Variables		Number (%)	Prescription rates (all medicine) %	Spearman’s rho	*P*-value
Contact type	Clinic consultations	1875 (79.3)	29.9		
	Home visits	488 (20.7)	16.2	0.125	< 0.001
Gender	Female	1073 (45.4)	26.7		
	Male	1290 (54.6)	27.3	0.006	0.77
Age (years)	< 1 year	642 (27.2)	20.2		
	1 year	627 (26.5)	30.5		
	2 years	376 (15.9)	31.6		
	3 years	282 (11.9)	24.1		
	4 years	235 (9.9)	34.0		
	5 years	201 (8.5)	25.4	−0.062	0.003
*GP-assessed*					
Temperature	No fever	1466 (62.0)	25.7		
	Unknown	6 (0.3)			
	Fever	891 (37.7)	29.2	0.038	0.064
Severity	Not sick	357 (15.1)	1.9		
	Not serious and unclear	1241 (52.5	27.3		
	Maybe serious	716 (30.3)	35.3		
	Clearly serious	49 (2.1)	4.1	0.136	< 0.001
Severity dichotomized	Not serious	1598 (67.6)	24.0		
	Possible/clearly serious	765 (32.4)	33.3	0.098	< 0.001
*Parental assessment*					
Duration of symptoms	Under 5 h	341 (27.9)	17.3		
(*n* = 1223)	5 to 12 h	238 (19.5)	34.0		
	More than 12 to 24 h	199 (16.3)	37.7		
	More than 24 h	429 (35.1)	30.5		
	Unknown	16 (1.3)	31.3	0.094	< 0.001
Severity (*n* = 1220)	Serious, possible life- threatening	59 (4.8)	18.6		
	Serious, but not life- threatening	663 (54.3)	29.7		
	Not serious or relevant	498 (40.8)	28.3	−.007	0.81
Severity Dichotomized (*n* = 1220)	Serious	722 (59.1)	28.8		
	Not serious or relevant	498 (40.8)	28.3	0.005	0.85
Satisfaction (*n* = 1221)	Very satisfied	540 (44.2)	33.0		
	Satisfied	463 (37.9)	27.2		
	Neutral	131 (10.7)	24.4		
	Dissatisfied	57 (4.7)	15.8		
	Very dissatisfied	28 (2.3)	14.3		
	Don’t know	2 (0.2)	0	0.085	0.003

### Reason for encounter

GPs recorded RFE according to the information provided by the parents during the consultation. About half the contacts (53%) had two RFEs: a primary and a secondary. Non-specific complaints, including fever and respiratory tract symptoms, were the most common primary symptoms; these were followed by skin symptoms and symptoms from digestive organs (Table 2). More than 70% of the secondary RFE belonged to another chapter than primary RFE. Fever was the primary or secondary RFE in 891 contacts (37.7, 95% CI: 35.8–39.7). Ear symptoms were present in 186 (7.9%, CI: 6.9–9.0) contacts; 119 of these had ear pain, corresponding to 5.0% (CI: 4.2–6.0) of all included contacts. In more than half the contacts (59.1%, CI: 56.4–61.9), the parents assessed the presented health problem as serious (Table 1), whereas the treating GPs assessed only 32.4% (CI: 30.5–34.3) of contacts to concern serious health problems. The symptoms had lasted for more than 24 h in about one-third of the contacts and for less than 5 h in 27.9% of contacts (CI: 25.4–30.5) (Table 1). We found symptoms of less than 5 h more often assessed as serious or maybe serious by the GPs (39.0%) compared to symptoms of longer duration (30.7%, diff. = 8.3%, CI: 3.5–13.1). The GPs did not state any RFE for 174 contacts (7%).

**Table 2:** Reason for encounter (primary and secondary) in 2363 preschool children at face-to-face consultations in out-of-hours primary care

RFE ICPC − 2	Primary RFE Consultation or home visit (% of children)	Secondary RFE Consultation or home visit	Total RFE
Unspecific complaints (Chapter A)	952 (40.3%)	351	1303 (55.1%)
Fever (A03)	673	218	891
Irritability (A16)	94	54	148
Unspecified illness (A99)	103	0	103
Respiratory tract (Chapter R)	553 (23.4%)	476	1029 (43.5%)
Coughing (R05)	239	206	445
Running nose (R07)	124	104	228
Dyspneu (R04)	55	58	113
Complain throat (R21)	49	39	88
Complain from Airways (R29)	21	9	30
Skin (Chapter S)	204 (8.6%)	62	266 (11.2%)
Exanthema (S06, S07)	94	48	142
Wound (S18)	45	0	45
Digestive organs (Chapter D)	192 (8.1%)	172	364 (15.4%)
Vomiting (D10)	66	73	139
Abdominal pain (D01)	35	10	45
Diarrhoea (D11)	29	39	68
Ear diseases (Chapter H)	102 (4.3%)	84	186 (7.8%)
Ear pain (H01)	58	61	119
Ear discharge ((H04)	16	14	30
Other ear complaints (H29)	14	1	15
Eye diseases (Chapter F)	33 (1.4%)	24	57 (2.4%)
Muscle and skeleton (Chapter L)	68 (2.9%)	20	88 (3.7%)
Nervous system (Chapter N)	15 (0.6%)	25	40(1.7%)
Urine tract (Chapter U)	25 (1.1%)	7	32(1.3%)
Endocrine organs (Chapter T)	15 (0.6%)	35	50(2.1%)
Male genitals (Chapter Y)	19 (0.8%)	3	22(0.9%)
Female genitals (Chapter X)	4 (0.2%)	1	5 (0.2%)
Other: blood, heart-vessel, psychiatric or social problems (Chapters B,K,P,Z)	7 (0.3%)	2	9 (0.4%)
Total	2189 (93%)	1262 (53%)	3451 (146%)
No RFE given	174 (7%)	–	–
Number of contacts	2363 (100%)		

Note: Secondary RFE was only recorded if it had another code or chapter number than the primary RFE

### Common diagnoses

Four percent of the contacts received two diagnoses, resulting in 2452 diagnoses for 2363 contacts (Table 3). The most common diagnosis was respiratory tract disease, which was present in 979 (41.4%, CI: 39.5–43.4); 360 of these were diagnosed as upper respiratory tract infection (RTI). General complaints, including fever and unspecified viral infection, was found in 438 (18.5%, CI: 17.0–20.2) of the diagnoses. Ear disease was the third-most common diagnosis group and was found in 370 (15.7%, CI: 14.2–17.2); 291 of these were diagnosed with acute otitis media (AOM).

**Table 3:** Diagnoses in 2363 OOH face-to-face consultations involving preschool children

Diagnosis ICPC-2	Number of consultations or home visits (% of all seen contacts)
Respiratory tract diseases (Chapter R)	979 (41.4%)
Upper respiratory tract infection (R74)	360
Acute bronchitis (R78)	147
Pneumonia (R81)	146
Tonsillitis/streptococcus tons (R76)	120
Laryngitis (R77)	74
General complaints (incl fever) (Chapter A)	438 (18.5%)
Viral infection, (A78)	157
Fever (A03)	84
Virus with exanthema (A76)	38
Ear diseases (Chapter H)	370 (15.7%)
Acute otitis media (H71)	291
Otitis media with effusion (H72)	45
Ear pain (H01)	23
Digestive organs (Chapter D)	237 (9.9%)
Gastroenteritis (D11)	101
Abdominal pain (D02)	37
Constipation (D12)	22
Skin diseases (Chapter S)	195 (8.3%)
Wound (S18)	46
Urticaria (S05)	20
Eye diseases (chapter F)	40 (1.7%)
Muscles/skeleton (Chapter L)	57 (2.4%)
Nerve diseases (Chapter N)	49 (2.1%)
Urine tract (Chapter U)	35 (1.5%)
Endocrine organs (Chapter T)	17 (0.7%)
Male genitals (Chapter Y)	20 (0.8%)
Female genitals (Chapter X)	8 (0.3%)
Other: blood, heart-vessel, psychiatric or social problems (Chapters B,K,P,Z) No diagnoses (*n* = 2)	7 (0.3%)
Total	2452 diagnoses in 2363 contacts

### Provided care

About three-quarters of the parents received some general advice from the GP. In 639 (27.0%, CI: 25.3–28.9) of the contacts, children were given one (601) or two (38) prescriptions of medicine. The prescriptions were less common at home visits (16.2%) compared with face-to-face consultations (29.9%) (Difference: 13.7%, CI: 16.2–29.9) (Table 1). Problem severity was related to prescription rates when assessed by the GPs (Rho = 0.136, *P* < 0.001). Short duration of symptoms was associated with a low prescription rate (17%). The prescriptions in the different age groups varied between 20 and 34% without any trend (Table 1).

The most common type of medication was oral antibiotics, which was prescribed 471 times (19.9%, CI: 18.4–21.6), and 33 children (1.4%) received topical antibiotics for eye infection (Table 4). In 222 contacts, children were prescribed penicillin-V (beta lactamase sensitive penicillin), corresponding to 47% of all prescribed oral antibiotics, and 216 (46%) were prescribed amoxicillin (beta lactamase resistant penicillin). No children received a prescription of cephalosporin or other newer broad-spectrum antibiotics. A total of 167 (7.1%, CI: 6.1–8.2) contacts involved prescriptions for other types of medicine than antibiotics, mostly for respiratory symptoms (*n* = 79). Of the 291 contacts ending with a diagnosis of AOM, 204 (70.1%, CI: 64.6–75.1) children were prescribed antibiotics. In 156 contacts (6.6%, CI: 5.7–7.7), children were prescribed antibiotics without receiving a diagnosis of fever or AOM. Most of these children (*n* = 89) were diagnosed with RTI (data not shown). Of the 891 contacts ending with a diagnosis of fever, 632 (70.9%, CI: 67.8–74.2) children received no prescription of antibiotics. In 374 contacts (15.8%, CI: 14.4–17.4), parents were advised to buy over-the-counter medicine, mainly paracetamol (Table 4), and 12% were recommended to make an appointment with their own GP.

**Table 4:** Prescriptions for 639 preschool children in 2363 face-to-face consultations in out-of-hours primary care

ATC-code	Name	Number	Percent of oral antibiotics
Oral antibiotics			
J01CA04	Amoxicillin	216	46%
J01CA08	Selexid	3	1%
J01 CE02	PenicillinV	222	47%
J01CF01	Inj.BenzylPenicillin	5	1%
J01CR02	Amoxicillin+Clavulanacid	5	1%
J01EA01	Trimetroprim	2	0.4%
J01 EB02	Sulfametizol	2	0.4%
J01FA01	Erytromycin	11	2%
J01FA09	Claritromycin	4	1%
J01FA10	Azithromycin	1	0.2%
Total oral antibiotics		471	100%
Topical antibiotics			
Eye creme S01AA01	Chloramphnicol	13	
S01AA13	Fucithalmic	20	
Ear drops S02AA, S02CA	Ciprofloxacin without and with hydrocortison,	12	
Group S (other)		4	
Group R (respiratory med.)		79	
Group D (digestic med.)		38	
Other prescriptions		40	
Total number of prescriptions		677^a^ In 639 contacts	
Over-the-counter medicine recommended		374	

Notes:^a^ In 38 contacts two prescriptions were made

### Referral

In 175 (7.4%, CI: 6.4–8.5) face-to-face consultations or home visits, children were referred for further evaluation or admission to a nearby hospital, mainly a paediatric department. Children under one year of age were more often referred (12%) than children over one year of age (6%). The diagnoses of the referred children were mainly respiratory diseases, such as bronchiolitis, pneumonia, or asthma (*n* = 97), or general bad condition, and these were often combined with high fever (*n* = 34) **(**Table 5). In 151 cases (86.3%, CI: 80.4–90.6), the GP considered the condition of the referred child to be serious or potentially serious.

**Table 5:** Diagnosis chapters of the 175 (7.4%) preschool children referred to hospital care from out-of-hours primary care (clinic consultations and home visits)

Chapter in ICPC-2	Number	Per cent
R (Respiratory tract)	97	55%
A (general problems, high fever)	34	19%
D (digestive organs, diarrhoea)	21	12%
N (neurological diseases)	11	6%
U (urinary system, cystitis)	9	5%
H (ear diseases, AOM)	8	5%
T (dehydration, no appetite)	7	4%
S (skin infections)	5	3%
Other	3	2%
Total number of diagnoses	195^a^	111%

Notes: ^a^20 children had two diagnoses

### Satisfaction

A total of 1003 (82.0%, CI: 79.8–84.1%) parents reported to be satisfied or very satisfied, 133 (10.9%, CI: 9.3–12.7) reported to be neutral or “unsure”, and 85 (7.0%, CI: 5.7–8.5) reported to be dissatisfied with the contact (Table 1). A low antibiotic prescription rate was associated with dissatisfied parents (Rho = 0.085, Table 1). Among the questionnaire respondents, 796 cases with a health problem rated as ‘not serious’ by the GP, 67 (8.4%, CI: 6.7–10.6) of parents were dissatisfied compared to only 18 of 425 (4.2%, CI: 2.7–6.6) of parents were dissatisfied when the health problem was rated as ‘serious’ by the GP. The parental satisfaction was higher when the child was referred, 90.5% compared to 81.4% (Chi-squared = 6.3, df. = 2, *p* = 0.042).

## Discussion

### Main findings

Non-specific complaints, including fever, were the most common primary or secondary RFE (1303, 55.1%) in our random sample of children 0–5 years of age seen by a GP at the OOH primary care service. Fever alone was identified in 891 (37.7%) children. Respiratory tract disease was the most common diagnosis group (41.4%); 360 (15.2%) had upper respiratory infection, 438 (18.5%) had general complaints, and 370 (15.7%) had ear diseases. A total of 639 (27%) contacts resulted in prescriptions, and 471 (20%) were prescribed antibiotics. In total, 70.1% of children with AOM received antibiotics, and 7.4% were referred for further examination/treatment at a paediatric or emergency department. In total, 7.0% of the parents reported that they were dissatisfied with the quality of the contact with the GP-run OOH service. Two percent of the children did not receive a diagnosis after being seen by a GP.

### Comparison with other studies

#### Use of OOH service

The original OOH organization, which was based on services provided by GPs for their own listed patients, has changed into large GP cooperatives with telephone triage, and regional clinics have become integral parts of the new model [1, 10]. In both the UK and Poland, children under five years of age have been found to have about fourfold more contacts with the OOH service than adults [11, 12]. Huibers et al. compared the use of OOH services in Denmark and the Netherlands. They found that Danish children had 250 contacts per 1000 inhabitants per year compared with Dutch children who had less than 100 contacts per 1000 inhabitants per year [4].

#### Reason for encounter (RFE)

Only few studies have reported RFE in children seen in OOH primary care, which makes comparison difficult [13]. A Dutch study based on a population including 20% under age five years reported that 25% of the parents contacted the OOH service with non-specific complaints; 15% were caused by respiratory problems in the child [14]. We found that non-specific complaints were the primary or secondary RFE in 1303 (55.1%) contacts, and complaints of respiratory tract symptoms were identified in 43.5%. These differences can be explained by different use of OOH primary care, telephone contacts included, and different age groups. In a Norwegian study from 2008, Welle-Nilsen et al. reported that one third of 210 OOH consultations concerned children aged 0–10 years. They found that 28% were classified with minor ailments; cough, fever, sore throat, upper RTI, and earache were the most common RFEs [15]. Another Norwegian study found fever was the most frequent RFE in children when nurses did telephone counselling [16]. De Bont et al. reported that 31% of contacts to a large Dutch GP OOH service concerning children under age 12 years were fever related [17]. This figure corresponds largely to our finding of 37.7% in a somewhat younger population.

#### Diagnosis

A multinational study exploring the diagnostic scope in OOH primary care in eight European countries found respiratory problems in 14–44% of children under the age of 18 years, general and non-specific complaints in 11–24%, and ear problems in up to 13% [3]. In an OOH paediatric clinic in the US, Goodrich et al. found that 26% of children under the age of 15 years presented with upper respiratory infection and 14% with otitis media or related conditions [18]. These figures are very similar to our findings although the age group investigated in our study was younger. Kozin et al. presented US figures on otology-related diagnoses given in an emergency department setting in 2009–11. They included children aged 0–17 years who presented with an ear complaint. In total, 82% were diagnosed with suppurative or unspecified otitis media; this corresponds to 5.6% of all visits [19]. This is in line with our finding of AOM in about 12% of younger children.

#### Provided care

Salisbury et al. found that 32% of all OOH primary care contacts in the UK ended with a prescription [11], and we found 27% in a Danish setting. Eishout et al. reported that 36.3% (CI: 31.3–41.7) of 322 febrile children (3 months - 6 years of age) seen by a GP in a face-to-face consultation in OOH primary care in the Netherlands were prescribed antibiotics [20]. We found that 25.0% (CI: 22.3–28.0) of the 891 contacts concerning children with fever ended with a prescription of antibiotics; this is significantly less, but the OOH service is more frequently used in Denmark than in the Netherlands [4]. In a Norwegian study with 401 children with respiratory symptoms and/or fever found prescription rate of antibiotics was 23% [21]. A C-reactive protein value over 20 mg/L, positive findings on ear examination, use of paracetamol and no vomiting were significant associated with antibiotic prescription.

In a population-based study of prescriptions of antibiotics during one year (2010–11) based on 644,777 OOH primary care contacts, Huibers et al. found that 25% of children (0–4 years of age) received an antibiotic prescription after a clinic consultation and 12% did after a home visit [22]. As this study was the basis for our study, the similar prescription rates (20% antibiotics and 7% other medicine) for children aged 0–5 years are not surprising.

We found that 16% of the children were diagnosed with ear-related problems and 12% with AOM; these results are in line with findings in Belgium and Spain [3]. The antibiotics prescription rate of 70% for contacts involving AOM in our study was lower than the figures reported from emergency department settings in the US, which has seen an increase from 79% in 1996 to 86% in 2004 [23].

#### Referrals

Giesen et al. analysed 4423 contacts to an OOH primary care service in the Netherlands for all age groups. They found that 7.1% were referred to an emergency department for further treatment [24]. In Norway Rebnord et al. found a referral rate of 7.7%. The strongest predictor for referral was affected respiration [21]. Shipman et al. found that 7.0% received a referral when contacting a cooperative OOH clinic in inner London [25]. They did not report any age stratification in the referral rates. We found a similar figure for preschool children in our study as 7.4% received a referral. However, we included only face-to-face consultations. As very few children are referred directly to hospital after a telephone contact, and face-to-face consultations accounted for 40.6% of all contacts in the CDR, the overall referral rate from OOH primary care is closer to 3% for all children.

#### Satisfaction

We acknowledge that the concept of satisfaction may be complex and several questions should optimally address what an assessment of patients’ experience of satisfaction is based on. However, we actually have several questions covering the patients’ experience of the encounter. In an earlier published paper, the issue of overall satisfaction has been addressed and found useful as it detects differences between groups of patients [26]. McKinley et al. reported in 1997 that 9.8% of the patients were dissatisfied with the OOH service when served by a GP and 17.9% when served by a deputising doctor [27]. In a study among 1139 respondents in Vejle County, which was conducted three years after the establishment of large-scale GP cooperatives in Denmark, Christensen et al. found that 13% were dissatisfied, 13% were neutral, and 74% were satisfied [1]. Our findings on parental satisfaction are in good agreement with this study as we found that 82% of the responding parents were satisfied, 11% were neutral, and 7.0% were dissatisfied. A study from Wales found that delays in response and triage times after using OOH services reduced the patient satisfaction, whereas a consultation length of over 10 min increased the satisfaction [28]. We have not identified other studies reporting on the correlation between low patient satisfaction and low prescription rates.

### Strengths and limitations

The study has several strengths. The random inclusion of children during the one-year study period minimised the risk of selection bias, and the one-year inclusion ensured that no seasonal bias existed in the data. Moreover, no drop-outs were observed in the electronic registrations. Our material of 2363 children (0–5 years) contacts is sufficiently sizeable to achieve high statistical precision.

It could be a limitation that our study counted contacts and not children. However, the risk of being included in the study more than once was less than 6% for patients seen in the OOH service during the one-year study period. It is a limitation that the diagnoses given rely exclusively on the individual GP’s clinical examination and evaluation of the child, in combination with the information provided by the parents. The low response rate for the postal questionnaire could have implied selection bias, and this potential risk must be considered when assessing the parental perceptions. The RFEs were based on the text stated by the GP in the medical record, and this text was subsequently ICPC-coded by a trained medical student and checked by one of the authors. This subsequent coding may have introduced a risk of misclassification as the stated text was sometimes ambiguous. The data could be considered a bit dated (2010–2011), but as no organisational changes have been made, results are expected to be valid.

#### Clinical implications and future research

The finding of 7.0% of parental dissatisfaction is in line of other studies on patient satisfaction, but additional studies seem relevant to identify reasons for dissatisfaction and further reduce dissatisfaction. Dissatisfaction may be related with the quality of communication and care in OOH service. The finding that some antibiotics were prescribed without a clear diagnosis points to the challenges that GPs face and the need for continuous awareness to limit unneeded antibiotic prescriptions. Whether a pediatrician or a GP should see children at OOH primary care services is not up for discussion in Denmark, because of the gatekeeping system with GPs taking care of children in daytime and outside office hours, without direct access to a pediatrician. Taking our results in account, one may deduct that a pediatrician is not needed. The diagnostic scope is most relevant for primary care and parents are satisfied with the care provided. Yet, this may be different in countries with another healthcare system, resulting in different patient expectations.

## Conclusions

We studied a random sample of face-to-face consultations and home visits at the OOH primary care service for 2363 contacts concerning children under the age of six years during one year with no drop-outs. The most common RFEs were non-specific complaints (40%) and respiratory tract symptoms (23%), whereas fever was identified in 38% of the contacts. The GPs diagnosed respiratory tract disease in 41% and ear disease in 16% and made a prescription for 27% of children (20% for systemic antibiotics). In total, 7.4% of children were referred to a hospital mostly for respiratory problems. Parental satisfaction was generally high, but 7.0% of the parents were dissatisfied with the contact; this needs further exploration.

## Abbreviations

AOM: acute otitis media
ATC: Anatomical Therapeutic Chemical Classification system
CDR: Central Denmark Region
CI: confidence interval
df: degree of freedom
GP: general practitioner
ICPC-2: International Classification of Primary Care, second edition
LV-KOS: population-based study of contact patterns in Danish out-of-hours primary care [Kontakt- og Sygdomsmønstre i LægeVagten]
OOH: out-of-hours
RFE: reason for encounter
RTI: respiratory tract infection
